# Argon inhibits reactive oxygen species oxidative stress via the miR-21-mediated PDCD4/PTEN pathway to prevent myocardial ischemia/reperfusion injury

**DOI:** 10.1080/21655979.2021.1965696

**Published:** 2021-09-10

**Authors:** Hong Qi, Jiancheng Zhang, You Shang, Shiying Yuan, Chunqing Meng

**Affiliations:** a Department of ICU, Union Hospital of Tongji Medical College of Huazhong University of Science and Technology, Wuhan, China; b Department of Orthopedic Surgery, Union Hospital of Tongji Medical College of Huazhong University of Science and Technology, Wuhan, China

**Keywords:** Argon, ROS oxidative, miR-21, PDCD4/PTEN pathway

## Abstract

The objective of this study was to explore the effect of argon preconditioning on myocardial ischemia reperfusion (MI/R) injury and its mechanism. Cardiomyocytes H2C9 were pre-treated with 50% argon, and a cell model of oxygen-glucose deprivation (OGD) was established. CCK-8 and cytotoxicity detection kits were used to detect cell viability and lactate dehydrogenase (LDH) release. The miR-21 expression was detected using quantitative real-time polymerase chain reaction. Western blot analysis was performed to detect the expression of programmed cell death protein 4 (PDCD4) and homologous phosphatase and tensin homolog (PTEN) proteins. The levels of inflammatory factors (IL-1β, IL-6, and IL-8) and oxidative stress factors (reactive oxygen species ROS], malondialdehyde [MDA], and superoxide dismutase [SOD]) were measured using an enzyme-linked immunosorbent assay. The effect of argon on cell apoptosis was detected using flow cytometry. Argon increased the proliferation of cardiomyocytes induced by OGD, decreased the release of LDH in cell culture medium, increased miR-21 expression in cells, decreased the expression of miR-21 target proteins PDCD4 and PTEN, decreased the levels of inflammatory factors (interleukin-1β [IL-1β], interleukin-6 [IL-6], and interleukin-8 [IL-8]) and oxidative stress factors (ROS and MDA), increased the SOD content, and decreased the cell apoptosis rate. Our results suggest that argon preconditioning inhibited the PDCD4/PTEN pathway via miR-21, thereby inhibiting ROS oxidative stress and preventing MI/R injury.

## Introduction

Acute myocardial infarction (AMI) is the main cause of sudden death worldwide and mainly manifests as ischemic necrosis caused by coronary artery occlusion [1]. Early reperfusion is a typical therapy of AMI, which can effectively reconstruct blood flow in ischemic myocardial tissue; however, reperfusion itself will increase irreversible damage to coronary artery circulation and accelerate and expand the myocardial ischemia reperfusion (MI/R) injury [2]. Myocardial protection during cardiac surgery has always been the focus of clinical and experimental research; however, there is no adequate response to date. Therefore, new interventional targets and adjuvant treatment methods must be found urgently to reduce heart reperfusion injury.

Recently, inert gas has become a research hotspot because of its potential organ protective effect. Argon, as one of the main components, has a protective effect on nerves and myocardium after ischemia reperfusion injury [3–6]. In vivo, 30% and 50% argon pre-treatment significantly reduced the apoptosis and inflammation level and improved the cell survival rate in human cardiomyocytes by AKT activation and differential regulation of MAP kinases [4]. Kiss et al. found that argon-mediated protection of myocardial tissue may be related to JNK and high-mobility group box 1 [5]. However, the molecular mechanism of argon protecting cardiomyocytes from ischemia reperfusion injury remains unclear.

MicroRNA (miRNA) is a type of endogenous non-coding RNA existing in eukaryotes and is approximately 18–25 nt in length. Previous studies have shown that microRNAs ameliorate MI/R injury [7–9]. Studies have confirmed that miR-21 expression is significantly reduced after MI/R, and that miR-21 overexpression can decrease the myocardial infarction area and cardiomyocyte apoptosis during chronic ischemia reperfusion injury [10,11]. Jia et al. found that xenon pre-treatment before ischemia reperfusion injury could induce up-regulation of miR-21 expression [12]. However, no studies have been conducted on the role of miR-21 in the inert gas protection of myocardium.

Previous studies have shown that MI/R injury is closely related to oxygen-free radical accumulation and mitochondrial dynamics disorder, affecting myocardial cell proliferation and apoptosis [13,14]. MiR-21 can repair MI/R injury by affecting reactive oxygen species (ROS)-mediated oxidative stress [15,16]. Programmed cell death protein 4 (PDCD4) is an important tumor suppressor that plays a vital role in cellular oxidative stress. Recent studies have shown that miR-21 expression is associated with PDCD4 expression and cardiomyocyte survival during cardiac protection against chronic oxidative stress [17]. Other studies have shown that homologous phosphatase and tensin homolog (PTEN) is the direct target of miR-21, and ROS can participate in cardiomyocyte apoptosis by regulating PTEN expression [18,19].

In the present study, we evaluated the protective effect of argon on ROS oxidative stress in MI/R injury and explored the underlying mechanisms involving the miR-21-PDCD4/PTEN axis. We speculated that argon might inhibit ROS oxidative stress by regulating the miR-21-PDCD4/PTEN axis, thereby preventing MI/R.

## Methods

### Cell culture

H2C9 cells were obtained from the American Type Culture Collection (USA) and stored in liquid nitrogen. The cells were cultured in Dulbecco’s modified Eagle’s medium containing 10% fetal bovine serum and placed in a cell incubator (MCO-20AIC, Panasonic, Japan) at 37°C and 5% CO_2_ for routine culture [20]. The cells were digested with 0.25% trypsin. Logarithmic growth phase cells were selected for the experiment.

## Oxygen glucose deprivation (OGD) cell model construction and grouping

The cells were divided into six groups. The normal group cells were cultured in an incubator containing 21% O_2_, 5% CO_2_, and 74% N_2_. The short-term hypoxia group and long-term hypoxia group cells were cultured in an incubator containing 1% O_2_, 5% CO_2_, and 94% N_2_ for 5 h and 10 h. The argon pre-treatment group cells were cultured with 50% argon for 1 h in advance and then placed in an incubator containing 21% O_2_, 5% CO_2_, and 74% N_2_. The argon + short-term hypoxia group and the argon + long-term hypoxia group cells were pre-treated with 50% argon for 1 h and then placed in an incubator containing 1% O_2_, 5% CO_2_, and 94% N_2_ for 5 h and 10 h. The cells and cell supernatants were collected 24 h after hypoxia (Supplementary Figure S1). The specific cell culture method was undertaken according to the methodology described in a previous study [21].

## Cell proliferation assay

A CCK-8 kit (Boster Biological, China) was used to detect cell proliferation following the procedure described in a previous study [22]. A cell suspension (100 µL/well) from each group was inoculated in 96-well plates. Then, 10 µL of CCK-8 reagent was added to each well at 24 h, 48 h, and 72 h. After that, all 96-well plates were placed in an incubator at 37°C for 2 h, and the spectrophotometric absorbance at 450 nm was recorded.

## Cell lactate dehydrogenase (LDH) determination

According to the manufacturer’s instructions, LDH release was measured quantitatively using a cytotoxicity test kit. A cell suspension (100 µL/well) from each group was inoculated in 96-well plates. The 96-well plate was centrifuged with a perforated plate centrifuge, the supernatant was discarded, and diluted LDH release reagent was added. After centrifugation, the supernatant from each well was added to the corresponding well of a new 96-well plate, and then the samples were determined with a HITACHI 7600–010 automatic biochemical analyzer.

## Quantitative real-time PCR (qRT-PCR)

Total RNA was isolated from the cells using Trizol reagent (Takara, Japan). The cDNA was reverse transcripted using an RNA reverse transcription kit (Thermo Fisher Scientific, USA). A SYBR Green RT-PCR kit (Takara) and ABI prism 7500 instrument (Applied Biosystems, USA) were used for qRT-PCR. The reaction conditions were pre-denaturation at 95°C for 3 min, 40 cycles at 95°C for 12 s and 62°C for 40 s, and extension at 72°C for 6 min. The primers were synthesized by SANGON Biotech (China): miR-21 forward 5′- CCG CTT CAA CAT CAG TCT GAT AAG CTA TTT TTT G-3′ and reverse 5′- AAT TCA AAA AAT AGC TTA TCA G-3′; GAPDH (internal control) forward, 5′-CCA GGT GGT CTC CTC TGA-3′ and reverse 5′-GCT GTA GCC AAA TCG TTG T-3′. The relative expression levels of the related genes were calculated using the 2^−ΔΔCt^ method [23].

## Western blot assay

Cells were collected and lysed with RIPA lysis buffer (Thermo Fisher Scientific). After centrifugation, the lysate was collected, and the protein concentration was determined using a BCA kit (Thermo Fisher Scientific). After sodium dodecyl sulfate-polyacrylamide gel electrophoresis (SDS-PAGE) electrophoresis, the protein was transferred to a polyvinylidene fluoride membrane and sealed in 5% bovine serum albumin (BSA) for 1 h. Then, it was incubated with a primary antibody at 4°C overnight. The antibody was purchased from Abcam (USA) and used at the dilution recommended by the manufacturer. After washing three times, the secondary antibody (Santa Cruz, USA) was incubated at 25°C for 1 h. Finally, an ECL kit was used to observe the protein bands. GAPDH was used as an internal reference.

## Enzyme-linked immunosorbent assay (ELISA)

The cell supernatant was collected, and the levels of inflammatory factors (interleukin-1β [IL-1β], interleukin-6 [IL-6], and interleukin-8 [IL-8]) and oxidative stress factors (ROS, superoxide dismutase [SOD], and malondialdehyde [MDA]) were detected using an ELISA kit (Abcam) according to the manufacturer’s instructions.

## Apoptosis analysis

The percentage of apoptotic cells at 72 h after transfection was determined with Annexin V-fluorescent-isothiocyanate (FITC)/propidium iodide (PI) double staining according to the manufacturer’s instructions [24]. After 0.25% trypsin digestion, the adherent cells were collected, and the cell density was adjusted to 1 × 10^6^/mL. After centrifugation, the cells were re-cultured with buffer solution and mixed gently with Annexin V-FITC/PI. The apoptosis rate of cardiomyocytes was detected using flow cytometry and analyzed with Cell Quest software.

## Statistical analysis

All experiments were repeated at least three times, and all data are expressed as mean ± standard deviation (SD). ne-way analysis of variance was used to compare the differences between groups. All statistical analyses were performed on GraphPad 7.0 software. Differences were considered statistically significant at *P* < 0.05.

## Results

The present study aimed to evaluate the protective effect of argon on ROS oxidative stress-induced MI/R injury, and the determination of the underlying mechanisms involving the miR-21-PDCD4/PTEN axis. A glyoxylate-deprived cell model was established and pre-treated with 50% argon to observe 1) cell proliferation and apoptosis; 2) miR-21, PDCD4, and PTEN expression; and 3) inflammation and oxidative stress. We speculated that argon might inhibit ROS oxidative stress by regulating the miR-21-PDCD4/PTEN axis, thereby preventing MI/R.

## Argon improved the proliferation ability of OGD-induced cells

**Figure 1.**
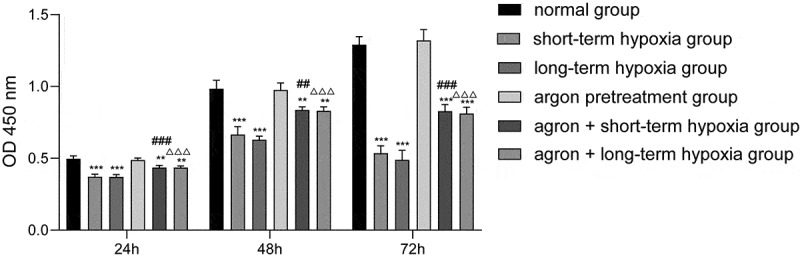
Effect of argon on the viability of OGD-induced cells. **P < 0.01, ***P < 0.001 *vs*. normal group; ^##^*P* < 0.01, ^###^*P* < 0.001 *vs*. short-term hypoxia group; ^ΔΔΔ^*P* < 0.001 *vs*. long-term hypoxia group

In the present study, CCK-8 was used to detect cell viability. The cell viability of the short-term hypoxia, long-term hypoxia, argon + short-term hypoxia, and argon + long-term hypoxia groups was significantly lower than that of the normal group (*P* < 0.001, Figure 1). The cell viability of the argon + short-term hypoxia group was significantly higher than that of the short-term hypoxia group (*P* < 0.001) and that of the argon + long-term hypoxia group was significantly higher than that of the long-term hypoxia group. Therefore, argon improved the proliferation of OGD cells (*P* < 0.001).

## Argon decreased LDH activity of OGD-induced cells

**Figure 2.**
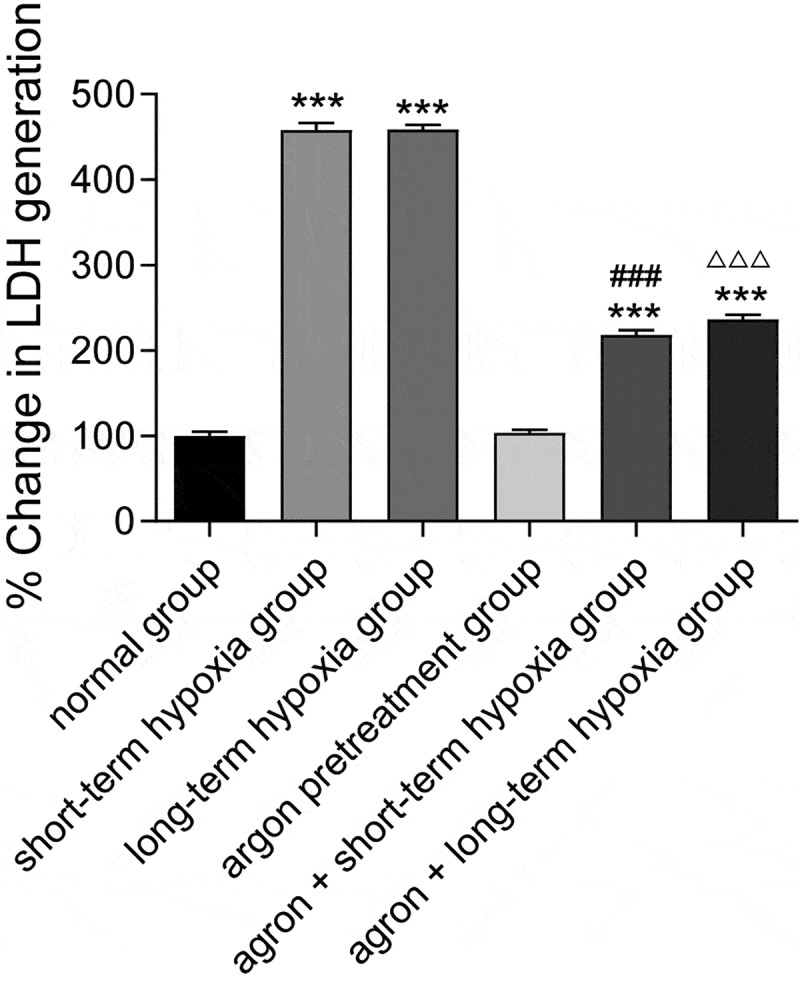
Effect of argon on LDH release in OGD-induced cells. ***P < 0.001 *vs*. normal group; ^###^*P* < 0.001 *vs*. short-term hypoxia group; ^ΔΔΔ^*P* < 0.001 *vs*. long-term hypoxia group

LDH is a stable cytoplasmic enzyme that exists in all cells. When the cell membrane is damaged, LDH is released quickly into the culture medium. In the present study, the release of LDH was significantly higher in the short-term hypoxia, long-term hypoxia, argon pre-treatment + short-term hypoxia, and argon pre-treatment + long-term hypoxia groups than that in the normal group (*P* < 0.001). The release of LDH in the argon + short-term hypoxia group was significantly lower than that in the short-term hypoxia group (218.10 ± 9.88 *vs*. 457.95 ± 14.72, *P* < 0.001). The release of LDH in the argon + long-term hypoxia group was significantly lower than that in the long-term hypoxia group (236.20 ± 9.82 *vs*. 458.5 ± 9.36, *P* < 0.001) (Figure 2). Therefore, argon protected cardiomyocytes from reperfusion injury.

## Argon increased miR-21 expression in OGD-induced cells

**Figure 3.**
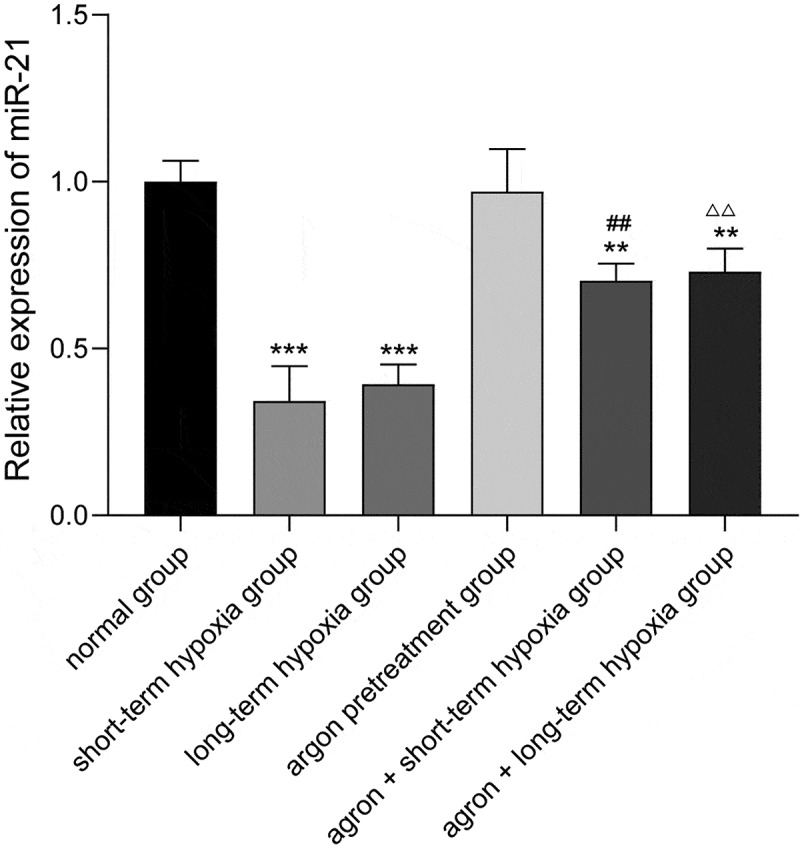
Effect of argon on miR-21 expression in OGD-induced cells. **P < 0.01, ***P < 0.001 *vs*. normal group; ^##^*P* < 0.01 *vs*. short-term hypoxia group; ^ΔΔ^*P* < 0.01 *vs*. long-term hypoxia group

The miR-21 expression level in each group was measured using qRT-PCR, and the results are shown in (Figure 3). The miR-21 expression level in the cells of the short-term hypoxia, long-term hypoxia, argon pre-treatment + short-term hypoxia, and argon pre-treatment + long-term hypoxia groups was significantly lower than that in the normal group (*P* < 0.01). The miR-21 expression level in the argon + short-term hypoxia group was significantly higher than that in the short-term hypoxia group (0.70 ± 0.05 *vs*. 0.34 ± 0.10, *P* < 0.01). The miR-21 expression level in the argon + long-term hypoxia group was significantly higher than that in the long-term hypoxia group (0.73 ± 0.07 *vs*. 0.39 ± 0.06, *P* < 0.01). Therefore, argon promoted miR-21 expression.

## Argon decreased miR-21 target protein expression in OGD-induced cells

**Figure 4.**
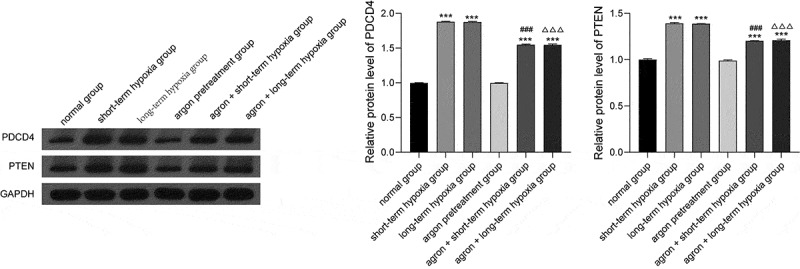
Effect of argon on the expression of PDCD4 and PTEN (miR-21 target proteins) in OGD-induced cells. ***P < 0.001 *vs*. normal group; ^###^*P* < 0.001 *vs*. short-term hypoxia group; ^ΔΔΔ^*P* < 0.001 *vs*. long-term hypoxia group

Many studies have shown that PDCD4 and PTEN are the target proteins of miR-21. In the present study, we measured PDCD4 and PTEN expression with western blot analysis. PDCD4 and PTEN expression levels in the short-term hypoxia, long-term hypoxia, argon + short-term hypoxia, and the argon + long-term hypoxia groups were significantly higher than that of the normal group (*P* < 0.001). The PDCD4 and PTEN expression levels were significantly lower in the argon + short-term hypoxia group than that in the short-term hypoxia group (*P* < 0.001). The PDCD4 and PTEN expression levels were significantly lower in the argon + long-term hypoxia group than that in the long-term hypoxia group (*P* < 0.001) (Figure 4). Therefore, argon affected the miR-21-mediated PDCD4/PTEN pathway.

## Argon decreased the expression of inflammatory factors and oxidative stress factors in OGD-induced cells

**Figure 5.**
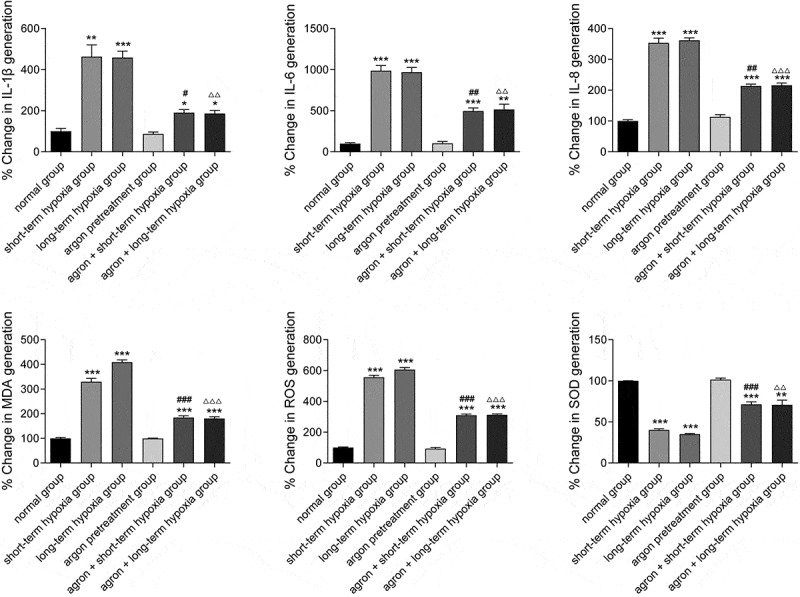
Effects of argon on the contents of inflammatory factors (IL1β, IL-6, and IL-8) and oxidative stress factors (ROS, MDA, and SOD) in OGD-induced cells. *P < 0.05, **P < 0.01, ***P < 0.001 *vs*. normal group; ^#^*P* < 0.05, ^##^*P* < 0.01 *vs*. short-term hypoxia group; ^###^*P* < 0.001 *vs*. short-term hypoxia group; ^ΔΔ^*P* < 0.01, ^ΔΔΔ^*P* < 0.001 *vs*. long-term hypoxia group

In the present study, the expression of inflammatory factors (IL-1β, IL-6, and IL-8) and oxidative stress factors (ROS, MDA, and SOD) in the cells of each group were measured using ELISA. The IL-1β, IL-6, IL-8, ROS, and MDA contents in the cells from the short-term hypoxia, long-term hypoxia, argon + short-term hypoxia, and argon + long-term hypoxia groups were significantly higher than those from the normal group, whereas the SOD content was significantly lower (*P* < 0.05, Figure 5). The IL-1β, IL-6, IL-8, ROS, and MDA contents in the argon + short-term hypoxia group were significantly lower than those in the short-term hypoxia group, whereas the SOD content was significantly higher (*P* < 0.01). The IL-1β, IL-6, IL-8, ROS, and MDA contents in the argon + long-term hypoxia group were significantly lower than those in the long-term hypoxia group, whereas the SOD content was significantly higher (*P* < 0.01). Therefore, argon inhibited oxidative stress and inflammatory factor expression via the miR-21-mediated PDCD4/PTEN pathway.

## Argon decreased the apoptosis of OGD-induced cells

**Figure 6.**
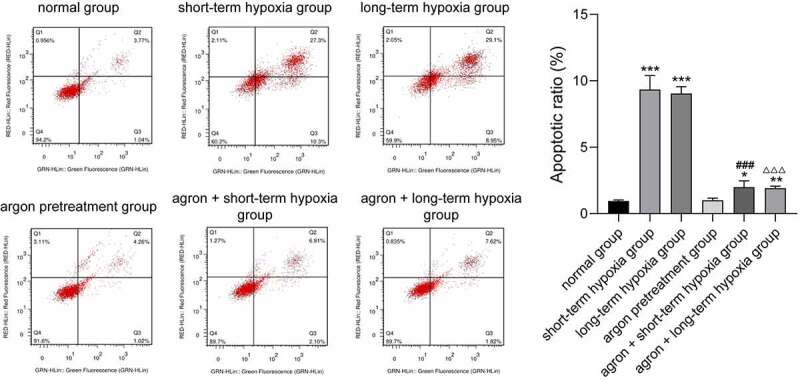
Effect of argon on the apoptosis of OGD-induced cells. *P < 0.05, **P < 0.01, ***P < 0.001 *vs*. normal group; ^###^*P* < 0.001 *vs*. short-term hypoxia group; ^ΔΔΔ^*P* < 0.001 *vs*. long-term hypoxia group

We detected the apoptosis rate in each group using flow cytometry. The apoptosis rates of cells in the short-term hypoxia, long-term hypoxia, argon + short-term hypoxia, and argon + long-term hypoxia groups were significantly higher than those in the normal group (*P* < 0.05, Figure 6). The apoptosis rate of cells in the argon + short-term hypoxia group was significantly lower than that in the short-term hypoxia group (1.99 ± 0.48 *vs*. 9.34 ± 1.05, *P* < 0.01). The apoptosis rate of the cells in the argon + long-term hypoxia group was significantly lower than that in the long-term hypoxia group (1.93 ± 0.11 *vs*. 9.04 ± 0.52, *P* < 0.01). Therefore, argon inhibited the apoptosis of OGD-induced cells via the miR-21-mediated PTEN/PDCD4 pathway.

## Discussion

Oxidative stress refers to the oxidative damage caused by the imbalance in production and elimination of oxygen-free radicals in the body or cells, resulting in ROS accumulation in the body or cells. ROS can be produced during MI/R, especially in the early reperfusion stage [25]. Excessive ROS can damage cardiomyocytes via various mechanisms, leading to cardiomyocyte apoptosis. Previous studies have shown that inert gas argon pre-treatment influences OGD-induced oxidative damage of human cardiomyocytes. Qi et al. reported that cardiomyocytes pre-treated with 30% argon for 90 min significantly increased cell activity and inhibited apoptosis [4]. In the present study, after pre-treatment with 50% argon for 1 h, the viability of cardiomyocytes induced by OGD increased and the apoptosis rate decreased significantly, which was similar to previous results. In addition, we found that argon decreased ROS and MDA contents and increased the SOD content. Therefore, argon activated the antioxidant pathway of cells and protected cardiomyocytes induced by OGD.

MiR-21, which is localized on chromosome 17, is a highly conserved endogenous small-molecule RNA binding to the 3′‐UTR region of target mRNA to repress target gene translation and inhibit target gene expression and is overexpressed in a variety of human tumor cells [26]. Recent studies have found that miR-21 is involved in the physiological and pathological processes of myocardial infarction, heart failure, cardiac hypertrophy, and other cardiovascular diseases [27–29].

Studies have confirmed that miR-21 expression is significantly reduced after ischemia reperfusion, and miR-21 overexpression decreases the myocardial infarct size and myocardial apoptosis during chronic ischemia reperfusion injury [10,11,30]. Cheng et al. found that transfection of miR-21 analogues attenuated H_2_O_2_-induced apoptosis of suckling mouse cardiomyocytes, and the antiapoptotic effect disappeared when miR-21 expression was inhibited [31]. In the present study, we found that argon pre-treatment significantly increased miR-21 expression in OGD-induced cardiomyocytes.

MI/R injury can lead to DNA fragmentation, mitochondrial damage, and eventual apoptosis, aggravating disease development [32]. Therefore, reducing cardiomyocyte apoptosis is of great significance in the treatment of MI/R injury. PDCD4 is a protein involved in apoptosis regulation [33]. Dong et al. demonstrated that 24 h after AMI, miR-21 significantly decreased the myocardial infarction size by the down-regulation of PDCD4 expression [34]. Xue et al. showed that miR-21 played a role in disease progression by down-regulating PTEN expression, which confirmed that PTEN might be a downstream target of miR-21 [35]. PTEN is a major negative regulator of the PI3K/AKT pathway, which plays an essential role in oxidative stress regulation [36]. PTEN negatively regulates the PI3K/AKT signaling pathway by dephosphorylating phosphatidylinositol triphosphate into phosphatidylinositol diphosphate and then influences cell apoptosis. The activation of PTEN/AKT signaling attenuates oxidative stress damage and reduces cardiomyocyte apoptosis after myocardial injury [37,38]. Our results showed that argon increased miR-21 expression, which inhibited PTEN and PDCD4 expression and reduced apoptosis in cardiomyocytes. We speculate that argon-induced miR-21 might relieve MI/R injury via inhibiting apoptosis regulated by the PI3K/AKT signaling pathway. The reduced oxidative stress regulated by PTEN-PI3K/AKT might also contribute to the mitigative effect of argon on MI/R injury.

## Conclusion

Argon preconditioning increased the viability of OGD-induced cardiomyocytes, reduced the release of LDH in cell culture medium, reduced the rate of cell apoptosis, and activated the antioxidant pathway in cells, which may be achieved via the miR-21-mediated PDCD4/PTEN pathway.

## Limitations

Although our findings provide a new theoretical basis for the treatment of MI/R injury, there remain some shortcomings of the present study: (1) the pathogenesis of MI/R injury is complex, and this study is only a preliminary exploration, (2) the findings are not supported by animal or clinical experimental data, and (3) the underlying mechanisms of PDCD4/PTEN action in MI/R have not been experimentally explored. Therefore, the potential action mechanisms of argon involving the miR-21-PDCD4/PTEN axis should be investigated in animal models of MI/R injury in the future.

## Supplementary Material

Supplemental Material

## Data Availability

All data generated or analyzed during this study are available from the corresponding author upon reasonable request.

## References

[1] Heusch G, Gersh BJ. The pathophysiology of acute myocardial infarction and strategies of protection beyond reperfusion: a continual challenge. Eur Heart J. 2017;38:774–784. 27354052 10.1093/eurheartj/ehw224

[2] Hausenloy DJ, Yellon DM. Targeting myocardial reperfusion injury–the search continues. N Engl J Med. 2015;373(11):1073–1075. 26321104 10.1056/NEJMe1509718

[3] Koziakova M, Harris K, Edge CJ, et al. Noble gas neuroprotection: xenon and argon protect against hypoxic-ischaemic injury in rat hippocampus in vitro via distinct mechanisms. Br J Anaesth. 2019;123(5):601–609. 31470983 10.1016/j.bja.2019.07.010 PMC6871267

[4] Qi H, Soto-Gonzalez L, Krychtiuk KA, et al. Pretreatment with argon protects human cardiac myocyte-like progenitor cells from oxygen glucose deprivation-induced cell death by activation of AKT and differential regulation of mapkinases. Shock. 2018;49(5):556–563. . 29658909 10.1097/SHK.0000000000000998

[5] Kiss A, Shu H, Hamza O, et al. Argon preconditioning enhances postischaemic cardiac functional recovery following cardioplegic arrest and global cold ischaemia. Eur J Cardiothorac Surg. 2018;54(3):539–546. . 29547976 10.1093/ejcts/ezy104

[6] Lemoine S, Blanchart K, Souplis M, et al. Argon exposure induces postconditioning in myocardial ischemia-reperfusion. J Cardiovasc Pharmacol Ther. 2017;22(6):564–573. . 28381122 10.1177/1074248417702891

[7] Sun M, Guo M, Ma G, et al. MicroRNA-30c-5p protects against myocardial ischemia/reperfusion injury via regulation of Bach1/Nrf2. Toxicol Appl Pharmacol. 2021;426:115637. 34217758 10.1016/j.taap.2021.115637

[8] Cheng C, Xu DL, Liu XB, et al. MicroRNA-145-5p inhibits hypoxia/reoxygenation-induced apoptosis in H9c2 cardiomyocytes by targeting ROCK1. Exp Ther Med. 2021;22(2):796. 34093752 10.3892/etm.2021.10228 PMC8170661

[9] Zheng T, Yang J, Zhang J, et al. Downregulated MicroRNA-327 attenuates oxidative stress-mediated myocardial ischemia reperfusion injury through regulating the FGF10/Akt/Nrf2 signaling pathway. Front Pharmacol. 2021;12:669146. 34025428 10.3389/fphar.2021.669146 PMC8138475

[10] Kim EN, Kim CJ, Kim SR, et al. High serum CRP influences myocardial miRNA profiles in ischemia-reperfusion injury of rat heart. PLoS One. 2019;14(5):e0216610. . 31063484 10.1371/journal.pone.0216610 PMC6504103

[11] Pan YQ, Li J, Li XW, et al. Effect of miR-21/TLR4/NF-kappaB pathway on myocardial apoptosis in rats with myocardial ischemia-reperfusion. Eur Rev Med Pharmacol Sci. 2018;22:7928–7937. 30536340 10.26355/eurrev_201811_16420

[12] Jia P, Teng J, Zou J, et al. miR-21 contributes to xenon-conferred amelioration of renal ischemia–reperfusion injury in mice. Anesthesiology. 2013;119(3):621–630. . 23681145 10.1097/ALN.0b013e318298e5f1 PMC4428598

[13] Luo X, Wu S, Jiang Y, et al. Inhibition of autophagy by geniposide protects against myocardial ischemia/reperfusion injury. Int Immunopharmacol. 2020;85:106609. 32446199 10.1016/j.intimp.2020.106609

[14] Wu J, Yang Y, Gao Y, et al. Melatonin attenuates anoxia/reoxygenation injury by inhibiting excessive mitophagy through the MT2/SIRT3/FoxO3a signaling pathway in H9c2 cells. Drug Des Devel Ther. 2020;14:2047–2060. 10.2147/DDDT.S248628 PMC7260543 32546969

[15] Climent M, Viggiani G, Chen YW, et al. MicroRNA and ROS crosstalk in cardiac and pulmonary diseases. Int J Mol Sci. 2020;21(12):4370. 10.3390/ijms21124370 PMC7352701 32575472

[16] Liu G, He L. Salidroside attenuates adriamycin-induced focal segmental glomerulosclerosis by inhibiting the hypoxia-inducible Factor-1alpha expression through phosphatidylinositol 3-Kinase/Protein kinase B pathway. Nephron. 2019;142(3):243–252. 30840958 10.1159/000497821

[17] Hao L, Wang J, Liu N. Long noncoding RNA TALNEC2 regulates myocardial ischemic injury in H9c2 cells by regulating miR-21/PDCD4-medited activation of Wnt/beta-catenin pathway. J Cell Biochem. 2019;120(8):12912–12923. 30861181 10.1002/jcb.28562

[18] Tu Y, Wan L, Fan Y, et al. Ischemic postconditioning-mediated miRNA-21 protects against cardiac ischemia/reperfusion injury via PTEN/Akt pathway. Plos One. 2013;8(10):e75872. . 24098402 10.1371/journal.pone.0075872 PMC3789724

[19] Wang XJ, Zhang MX, Chen XL. Silencing PTEN gene inhibits cardiomyocyte injury induced by H_2O_2 in rats. Basic Clin Med. 2019;39(05):705–709.

[20] Zhao G, Zhang X, Wang H, et al. Beta carotene protects H9c2 cardiomyocytes from advanced glycation end product-induced endoplasmic reticulum stress, apoptosis, and autophagy via the PI3K/Akt/mTOR signaling pathway. Ann Transl Med. 2020;8(10):647. 32566584 10.21037/atm-20-3768 PMC7290636

[21] Hafner C, Qi H, Soto-Gonzalez L, et al. Argon preconditioning protects airway epithelial cells against hydrogen peroxide-induced oxidative stress. Eur Surg Res. 2016;57(3–4):252–262. . 27560977 10.1159/000448682

[22] Tang C, Feng W, Bao Y, et al. Long non-coding RNA TINCR promotes hepatocellular carcinoma proliferation and invasion via STAT3 signaling by direct interacting with T-cell protein tyrosine phosphatase (TCPTP). Bioengineered. 2021;12(1):2119–2131. 34057016 10.1080/21655979.2021.1930336 PMC8806792

[23] Livak KJ, Schmittgen TD. Analysis of relative gene expression data using real-time quantitative PCR and the 2(-Delta Delta C(T)) Method. Methods. 2001;25(4):402–408. 11846609 10.1006/meth.2001.1262

[24] Yin G, Zeng Q, Zhao H, et al. Effect and mechanism of calpains on pediatric lobar pneumonia. Bioengineered. 2017;8(4):374–382. . 27786573 10.1080/21655979.2016.1234544 PMC5553339

[25] Neil Granger D, Kvietys PR. Reperfusion injury and reactive oxygen species: the evolution of a concept. Redox Biol. 2015;6:524–551. 26484802 10.1016/j.redox.2015.08.020 PMC4625011

[26] Hermansen SK, Dahlrot RH, Nielsen BS, et al. MiR-21 expression in the tumor cell compartment holds unfavorable prognostic value in gliomas. J Neurooncol. 2013;111(1):71–81. 23104517 10.1007/s11060-012-0992-3

[27] Li L, Chen Q, Feng C, et al. Aberrant expression of TNRC6a and miR-21 during myocardial infarction. 3 Biotech. 2019;9(7):285. 10.1007/s13205-019-1812-7 PMC6592996 31245249

[28] Duygu B, Da Costa Martins PA. Da costa martins PA. miR-21: a star player in cardiac hypertrophy. Cardiovasc Res. 2015;105(3):235–237. 25644540 10.1093/cvr/cvv026

[29] Cardin S, Guasch E, Luo X, et al. Role for MicroRNA-21 in atrial profibrillatory fibrotic remodeling associated with experimental postinfarction heart failure. Circ Arrhythm Electrophysiol. 2012;5(5):1027–1035. . 22923342 10.1161/CIRCEP.112.973214

[30] Huang J, Qi Z. MiR-21 mediates the protection of kaempferol against hypoxia/reoxygenation-induced cardiomyocyte injury via promoting Notch1/PTEN/AKT signaling pathway. PLoS One. 2020;15:e0241007. 33151961 10.1371/journal.pone.0241007 PMC7644004

[31] Cheng Y, Liu X, Zhang S, et al. MicroRNA-21 protects against the H(2)O(2)-induced injury on cardiac myocytes via its target gene PDCD4. J Mol Cell Cardiol. 2009;47(1):5–14. 19336275 10.1016/j.yjmcc.2009.01.008 PMC3593965

[32] Bello-Klein A, Khaper N, Llesuy S, et al. Oxidative stress and antioxidant strategies in cardiovascular disease. Oxid Med Cell Longev. 2014;2014:678741. 25031784 10.1155/2014/678741 PMC4086346

[33] Göke A, Göke R, Knolle A, et al. DUG is a novel homologue of translation initiation factor 4G that binds eIF4A. Biochem Biophys Res Commun. 2002;297(1):78–82. . 12220511 10.1016/s0006-291x(02)02129-0

[34] Dong S, Cheng Y, Yang J, et al. MicroRNA expression signature and the role of microRNA-21 in the early phase of acute myocardial infarction. J Biol Chem. 2009;284(43):29514–29525. . 19706597 10.1074/jbc.M109.027896 PMC2785585

[35] Xue X, Liu Y, Wang Y, et al. MiR-21 and MiR-155 promote non-small cell lung cancer progression by downregulating SOCS1, SOCS6 and PTEN. Oncotarget. 2016;7(51):84508–84519. . 27811366 10.18632/oncotarget.13022 PMC5356677

[36] Lee YR, Chen M, Pandolfi PP. The functions and regulation of the PTEN tumour suppressor: new modes and prospects. Nat Rev Mol Cell Biol. 2018;19(9):547–562. 29858604 10.1038/s41580-018-0015-0

[37] Yu L, Li Z, Dong X, et al. Polydatin protects diabetic heart against ischemia-reperfusion injury via Notch1/Hes1-mediated activation of Pten/Akt signaling. Oxid Med Cell Longev. 2018;2018:2750695. 29636838 10.1155/2018/2750695 PMC5831600

[38] Xu J, Tang Y, Bei Y, et al. miR-19b attenuates H2O2-induced apoptosis in rat H9C2 cardiomyocytes via targeting PTEN. Oncotarget. 2016;7(10):10870–10878. . 26918829 10.18632/oncotarget.7678 PMC4905445

