# Supplementary figures and images for: Argon inhibits reactive oxygen species oxidative stress via the miR-21-mediated PDCD4/PTEN pathway to prevent myocardial ischemia/reperfusion injury
Source: Bioengineered. 2021 Sep 10;12(1):5529–39. doi: 10.1080/21655979.2021.1965696 (PMC8806883; doi:10.1080/21655979.2021.1965696)

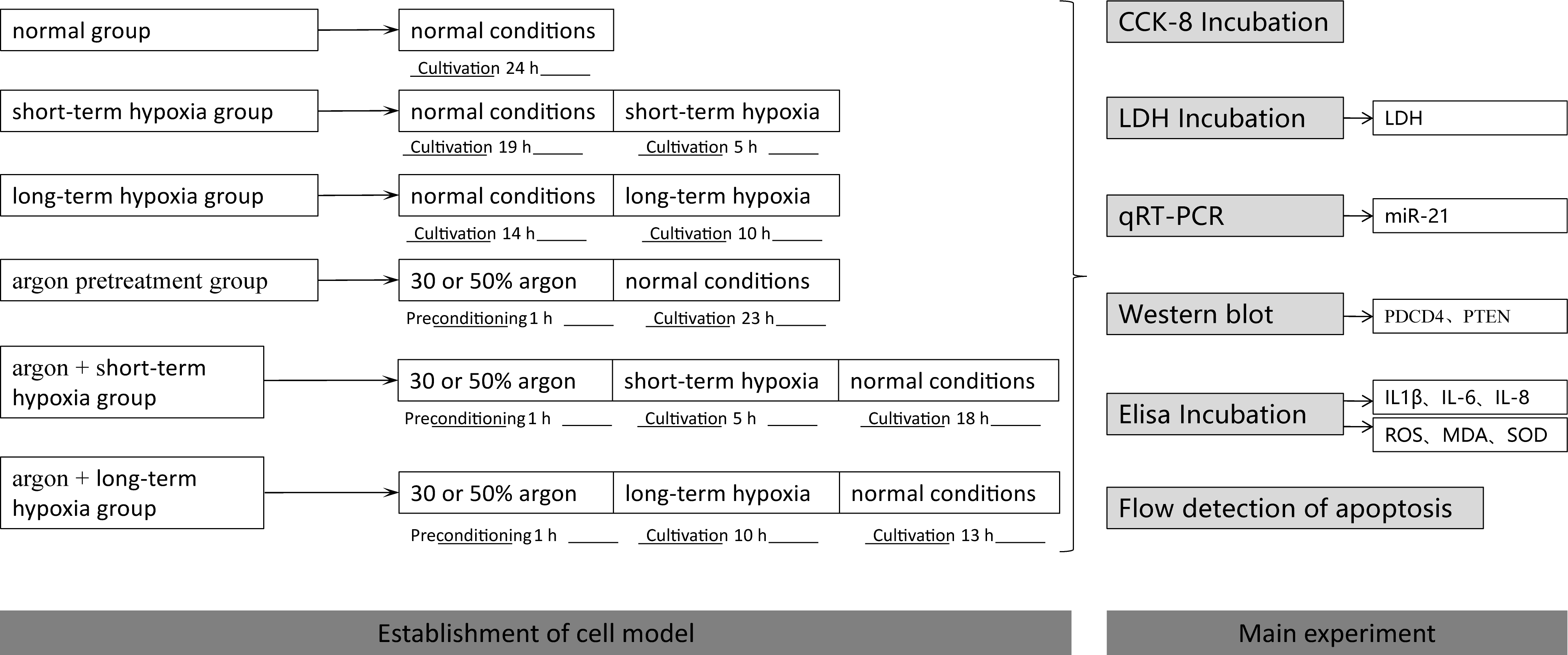

Supplement: Supplemental Material [file KBIE_A_1965696_SM6477.zip › Supplementary Figure S1.tif]
